# Implementation of a MEIoT Weather Station with Exogenous Disturbance Input

**DOI:** 10.3390/s21051653

**Published:** 2021-02-27

**Authors:** Héctor A. Guerrero-Osuna, Luis F. Luque-Vega, Miriam A. Carlos-Mancilla, Gerardo Ornelas-Vargas, Víctor H. Castañeda-Miranda, Rocío Carrasco-Navarro

**Affiliations:** 1 Unidad Académica de Ingeniería Eléctrica, Universidad Autónoma de Zacatecas, Zacatecas 98000, Mexico; hectorguerreroo@uaz.edu.mx (H.A.G.-O.); ornelas@uaz.edu.mx (G.O.-V.); vhcast@gmail.com (V.H.C.-M.); 2 Centro de Investigación, Innovación y Desarrollo Tecnológico CIIDETEC-UVM, Universidad del Valle de México, Tlaquepaque 45601, Jalisco, Mexico; miriam.carlos@uvmnet.edu; 3 Department of Mathematics and Physics, ITESO AC, Tlaquepaque 45604, Jalisco, Mexico; rociocarrasco@iteso.mx

**Keywords:** sensing system, internet of things, educational mechatronics, engineering education, hands-on learning

## Abstract

Due to the emergence of the coronavirus disease (COVID 19), education systems in most countries have adapted and quickly changed their teaching strategy to online teaching. This paper presents the design and implementation of a novel Internet of Things (IoT) device, called MEIoT weather station, which incorporates an exogenous disturbance input, within the National Digital Observatory of Smart Environments (OBNiSE) architecture. The exogenous disturbance input involves a wind blower based on a DC brushless motor. It can be controlled, via Node-RED platform, manually through a sliding bar, or automatically via different predefined profile functions, modifying the wind speed and the wind vane sensor variables. An application to Engineering Education is presented with a case study that includes the instructional design for the least-squares regression topic for linear, quadratic, and cubic approximations within the Educational Mechatronics Conceptual Framework (EMCF) to show the relevance of this proposal. This work’s main contribution to the state-of-the-art is to turn a weather monitoring system into a hybrid hands-on learning approach thanks to the integrated exogenous disturbance input.

## 1. Introduction

Technological advancement derived from more than 50 years of Moore’s Law has brought humanity into an era where the life cycles of products and technologies have been shortened. This advancement has also led to the knowledge generated by humanity doubling roughly every 2 years. For their part, educational systems are in a constant search for the adoption of new educational technologies that enhance their students’ education, preparing them for the skills that will be required in the jobs of the new industrial era. The transformation of educational systems has been slow in most educational institutions. However, with the emergence of the coronavirus disease 2019 (COVID 19), education systems in most countries accelerated this adoption and quickly changed the teaching strategy to online teaching [1,2,3,4,5]. Some of these works propose methodologies that integrate skills and attitudes towards this new form of education. However, one of the main challenges is when a hands-on learning (HOL) approach is involved. HOL in engineering education traditionally has involved students interacting with an artifact in a laboratory [6,7]. The combination of HOL and the COVID 19 situation has accelerated the necessity to develop technology that allows this interaction to be done remotely. Years before the emergence of COVID 19, there had already been proposals for remote experiments or online laboratories such as [8] that presented a remote control system that allows experiments to be carried out over the Internet. Yeung et al. [9] proposes a remote laboratory in real-time based on Supervisory Control And Data Acquisition (SCADA), with the control of an induction motor as an example and focused on making the laboratory environment accessible to multiple users. Similarly, Aydogmus et al. [10] presents a remote access laboratory with a distributed modular design to develop and share peer-to-peer (P2P) experiments. In [11], University of Malaysia Perlis (UniMAP) researchers present the development of a graphical interface accessible from the Internet that allows connection to some equipment in the laboratories to support students who could not attend laboratory work.

The literature presents a great variety of weather stations and climate monitoring devices developments. In reference [12], they integrate sensors with an Arduino Mega 2560 board and show the current data while generating a record of the sensors. In reference [13], they also implement a meteorological station connected through the Narrowband Internet of Things network and whose data is displayed using Grafana in any Internet browser. Parvez et al. [14], shows the development of a low-cost autonomous weather station for developing countries. Adepoju et al. [15] shows an Arduino-based weather station that sends the data in real-time to a central station. In reference [16], they present the development of a meteorological station that can transmit data through different communication protocols while maintaining a local record. Weather stations serve different purposes and applications in the literature. However, few weather stations are developed for educational purposes like the one proposed in reference [17].

A disturbance is an event that can affect the output of a system; these disturbances can be endogenous or exogenous according to the origin of the event. External disturbances, in particular, are used in different models in order to design more robust controllers, make the process of entering neural networks more efficient, etc.; these apply in several areas such as environmental [18,19,20], economy [21,22], control [18,22,23,24] and chemistry [23,24], to mention some.

Although the integration of exogenous disturbance inputs in models is well established [18,19,20,21,22,23,24,25,26], and in control applications, these exogenous disturbance inputs are generated to test controllers as in [27,28,29], applications that manipulate these inputs to affect the model in the modeling or capture stage are difficult to find. For some monitoring applications where studied variables have a natural low change rate, such as climate variables, an interesting approach would be to generate a controlled exogenous disturbance to accelerate the change rate of the monitored variables in order to obtain a better understanding of the variables range and their interactions. Such exogenous disturbances can be generated physically with an adequate actuator and an open-loop controller.

This combination of facts leads us to the following research questions: how could a weather station, which by design only monitors variables, be repurposed for hands-on usage to enhance the learning experience? How could this experience be transmitted to the students, who could not be physically present due to COVID 19, to interact with such weather stations? How could an educational framework be of help?

Hence, this work presents a weather station paired with a controlled exogenous disturbance in the context of an educational mechatronics framework that is adapted to a remote laboratory operation to give students an online HOL experience.

The paper is organized as follows. Section 2.2 presents the National Digital Observatory of Smart Environments (OBNiSE, by its initials in Spanish) architecture for educational mechatronics. In Section 3, the detailed description of the design and implementation of MEIoT weather station with exogenous disturbance input is presented. Section 4 shows the application to Engineering Education within the EMCF using the MEIoT weather station with exogenous disturbance input. Finally, Section 5 includes the discussion and provides directions for potential future ideas.

## 2. Materials and Methods

This section details the methodology used in this research work and the proposed software architecture used for educational mechatronics. It is worthwhile to mention that the development of the implementation of an exogenous disturbance input for the MEIoT weather station will be carried out in Section 3.

### 2.1. Methodology

The research presented in this work is an applied research that aims to develop an educational IoT device and a complete platform to offer an alternative for conducting a HOL approach in a remote access laboratory or hybrid education environment in the context of pandemic COVID-19. We collected the primary data directly to manipulate and control variables to determine cause and effect through a quantitative analysis focused on measuring, modifying, and interpreting the weather station variables’ behavior. The aforementioned was carried out with a flexible design developed through a real data acquisition process within the OBNiSE architecture focused on applying educational mechatronics described in the following section. Moreover, an instructional design based on the educational mechatronics conceptual framework is presented to demonstrate how to use the collected real data and interact with the MEIoT weather station through a remote Laboratory.

### 2.2. OBNiSE Architecture for Educational Mechatronics

The imminent change in processes since the appearance of the fourth industrial revolution, also called Industry 4.0, has led to the creation of projects and processes based on the Internet of Things (IoT). The IoT allows to improve and create new systems and architectures according to the new requirements of this Industry 4.0. These technological changes have allowed this pandemic not to be an obstacle, especially for education, which has adapted to a model of one hundred percent virtual classes that allow students to learn through distance learning strategies.

Several proposals have allowed educational institutions to adapt quickly to these changes. Among the most important works are those that use architectures or software-based on IoT. For instance, the IoT architecture called OBNiSE presented in [17] which is extended in this proposal to incorporate the educational component and describe the interaction of the elements in the architecture. Through OBNiSE layered composition, the description of any IoT system or application can be done from the integrated devices to the user application.

As it was described in the previous paper, this architecture is composed of six layers (1) A device layer that contains all devices to collect information. (2) A network layer that manages tools, cables, and users. (3) A processing layer in charge of information processing and visualization of data. (4) A cloud layer that ensures the availability of information to users, devices, and applications. (5) An application layer allows the connection of services, applications, and systems through mobile devices. Finally, (6) a security layer supervises all layers allowing the secure data transmission and secures the information of the users within the systems. For more information please consult [17].

This proposal presents the OBNiSE architecture applied to education, combining the Internet of Things and Educational Mechatronics. This architecture is based on IoT and comprises layers; each layer is responsible for the functionality of a complete system or mobile application and contains tools, devices, and a web service.

**Figure 1:**
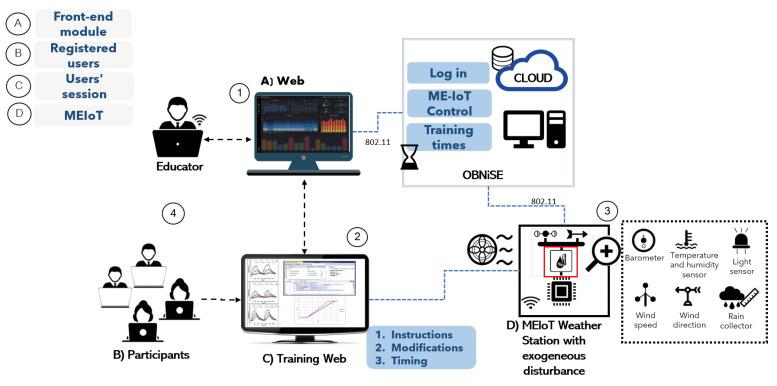
National Digital Observatory of Smart Environments (OBNiSE) Internet of Things (IoT) architecture for Educational Mechatronics.

The OBNiSE architecture inspires the proposed IoT Educational mechatronics architecture in this section. The proposed architecture is presented in Figure 1.

The OBNiSE IoT architecture for Educational Mechatronics involves several elements that interact at different levels of the system. For example, a Web system that includes data visualization from the MEIoT weather station’s sensors, a training web that is part of the web, a MEIoT weather station with different data sensors, and users that are divided into two categories educators and participants. Each of them is described in detail below.

1. Web system. It is hosted in a cloud system, which can be accessed by educators. The web shows all the sensors’ parameters and data that can be manipulated, the graphs of the real-time sensed data, the configuration of the MEIoT weather station, and the users’ training times per session. This web system is stored in the OBNiSE.
2. Traning web. It is part of the central web system. However, it has limitations for the participants’ view since it only allows them to view the MEIoT weather station’s current information but does not allow to change its configurations. The training web updates the information sent by the participants in real-time.
3. MEIoT weather station. It is an IoT device that integrates temperature, relative humidity, barometric pressure, altitude, light, rainfall, wind speed, and wind direction sensors. It is worthwhile to mention that this work proposes the inclusion of an exogenous disturbance input for the wind speed and the wind vane sensors; this allows the modification and observation of the system’s behavior itself.
4. Users. The system is composed of two types of users described as follows.
  - Educators. They have access to all the data and configuration of the MEIoT station and have the complete knowledge for handling the MEIoT station. Likewise, they allow or deny access to registered participants in the system. Educators also can modify the dashboard, data, time of actualization, among others.
  - Participants. These are students and people who are part of the mechatronics education course and require access to the platform to access the MEIoT weather station. The participants have limited access and only can modify some parameters allowed by the Educator for a defined time.

The MEIoT weather station records the modifications made in user sessions directly from the Web system connected to the cloud and allows users to graph the data. As already mentioned at the beginning of this section, the architecture presented respects the OBNiSE architecture interactions.

An exogenous disturbance input is introduced to modify the wind speed to provoke changes in the climatic station’s behavior; this variable can be modified from a web, manually or with different profiles, to which participants will have access to do so. For this first stage, only this variable is considered, however, it is planned that in the near future, all the MEIoT weather station sensors’ variables can be perturbed in a desirable manner with several exogenous disturbance inputs.

Finally, when working with multiple simultaneous user sessions on the web, a queuing system is managed, in which each request or change is attended in a *t* time determined by the Educator, to control the requests made during a total time *tt* established time per session. During active sessions, each participant can make one or more changes to the exogenous disturbance input causing changes in the wind speed variable and view the changes in the updated variable. This change will be displayed until a new input is considered.

## 3. Implementation of Exogenous Disturbance Input to the MEIoT Weather Station

A set of actions between the user and system is considered in the design stage; these actions can be for testing or education purposes. As already mentioned at the beginning of this work, this proposal is focused on an educational environment adapted to the newly emerging technological needs due to the pandemic in which students require tools that allow them to acquire new knowledge of industry 4.0 with techniques of education 4.0.

**Figure 2:**
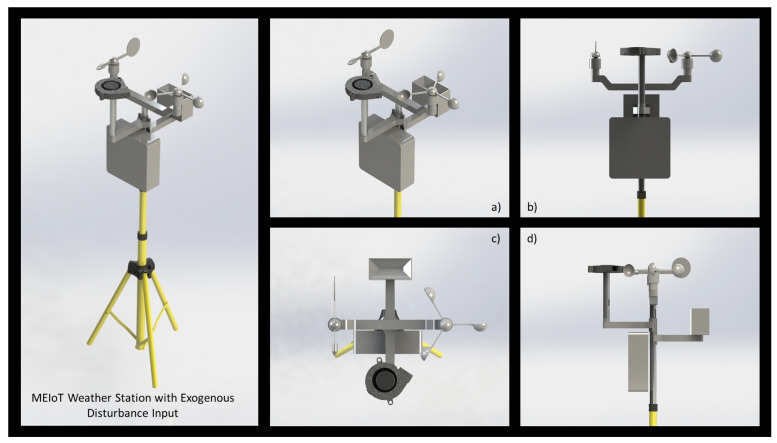
3D model design of the MEIoT weather station with exogenous disturbance input. (**a**) Isometric view, (**b**) front view, (**c**) top view, and (**d**) right view.

As described in [17], the MEIoT weather station comprises temperature, relative humidity, barometric pressure, altitude, light, rainfall, wind speed, and wind direction sensors, and a wind blower actuator was added to this work to generate an external disturbance while it is prepared to integrate more exogenous disturbances inputs. This disturbance is under the user control. The 3D model of the complete MEIoT weather station with an exogenous disturbances input can be seen in Figure 2.

More details about the MEIoT weather station with exogenous disturbance input implementation can be found in the following subsections.

### 3.1. The MEIoT Weather Station with Exogenous Disturbance Input

Implementing the exogenous input to the MEIoT Weather Station converts the device in a facility with instruments and equipment for measuring atmospheric conditions and a set of manipulable exogenous disturbance sources. This information can be used for different purposes.

**Figure 3:**
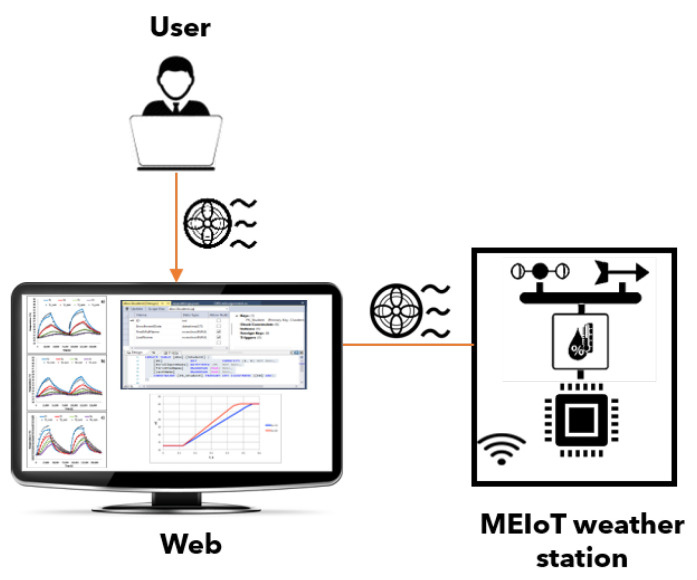
Participants and the exogenous disturbances.

As shown in Figure 3; participants and educators can modify entries to the MEIoT weather station using the Web system to monitor the sensors’ behavior, providing learning for students. The 3D model of the MEIoT Weather Station with exogenous disturbance input is shown in Figure 2.

### 3.2. Implementation of the MEIoT Weather Station with Exogenous Disturbance Input within the OBNiSE Architecture

**Figure 4:**
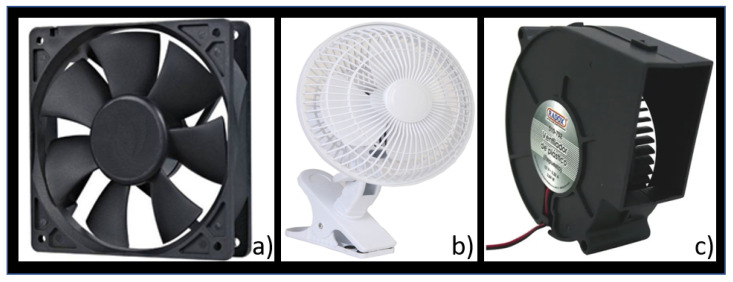
Wind sources as the exogenous disturbance input. (**a**) Computer fan, (**b**) mini desktop fan, (**c**) air blower.

**Figure 5:**
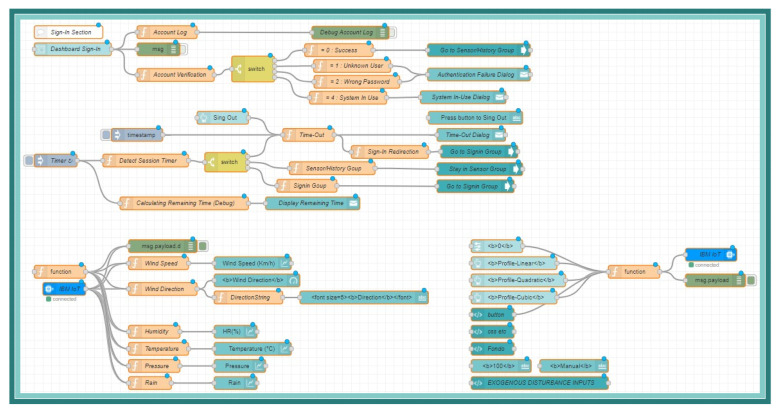
Node-Red based application.

According to the architecture proposed in [17], which is composed of 6 layers (Devices, Network, Processing, Cloud, Applications, Security), it is crucial to have an architecture that meets the requirements of industry 4.0 with elements of the Internet of things and that in turn complies with security, adaptability, robustness, and availability characteristics. That is why this architecture proposal includes the elements described in the OBNiSE architecture and extends the elements presented in [17] to strengthen the educational part through an application and an environment for students with hybrid learning. These elements are integrated into the OBNiSE platform as follows:

- Device Layer: The MEIoT weather station with exogenous disturbance input uses a microcontroller and a set of sensors described in [17] and a wind blower based on a DC brushless motor; described later in Section 3.3 and it is shown in Figure 4.
- Network Layer: This layer considers tools, user profiles, and data accessibility and ensures communication between the architecture’s devices and layers. The H-Bridge for actuating the blower is connected through a microprocessor dedicated Pulse Width Modulator (PWM) pin port with two digital signals for direction and enabling. The network layer is integrated by three elements: tools, user profiles, and data accessibility. Each item is composed as follows:
  - Tools: This item encompasses every tool required to connect the MEIoT weather station with its sensors, actuators, and application, from cables and connectors to the Virtual Network Computing (VNC). Any additional tool is also part of this element.
  - User profiles: Participants and educators were the two user-profiles defined. Participants can see the sensor’s information using a PC or a mobile and manipulate the actuator output; educators, on the other hand, also have the possibility of modifying parameters within the MEIoT weather station; these profiles are explained in Section 3.4.
  - Data accessibility. This element defines the communication channel for the devices, users, and information. The used protocols are WiFi and the Message Queuing Telemetry Transport (MQTT) protocol for this implementation.
- Processing Layer.The MEIoT weather station uses an ESP32 microprocessor with built-in WiFi capability to capture sensors’ data, drive actuators, and communicate to the cloud. IBM’s Watson IoT platform, a specialized platform for IoT, is employed for cloud computing.
- Cloud Layer. The information and data are stored using a database in the cloud; a Node-Red based application, Figure 5, manages access to it. The data are available for the MEIoT weather station, the web system, and the OBNiSE architecture. The IBM Watson platform is currently used for information storage.
- Applications Layer: For data visualization and exogenous disturbance input manipulation, a web application based on Node-RED was created.This application allows interaction with the MEIoT weather station and its exogenous disturbance input from a mobile device, computer, or the web. Section 3.4 explains it in more detail.
- Security is used across devices, applications, data storage, network, and processing layers. The security allows only the Educator profile to modify, configure, and see the complete information about the MEIoT weather sensors and register participants who can enter to see the system’s behavior. It is necessary to define modifications in the configurations for the data protection, the use of the application at the user profile and application levels, and the processing and the cloud data storage. The security implementation starts with the Watson IoT Platform, where unique organization IDs are assigned. IBM’s Watson IoT Platform has three main security aspects, Transport Layer Security (TLS), authentication, and authorization. These aspects remain the same as in [17]. Watson IoT platform integrates two kinds of policies: connection and messaging policies that provide access control using a Client ID and a User ID. Access is allowed as long as these credentials are valid; the verification is done through the MTTQ protocol.

**Figure 6:**
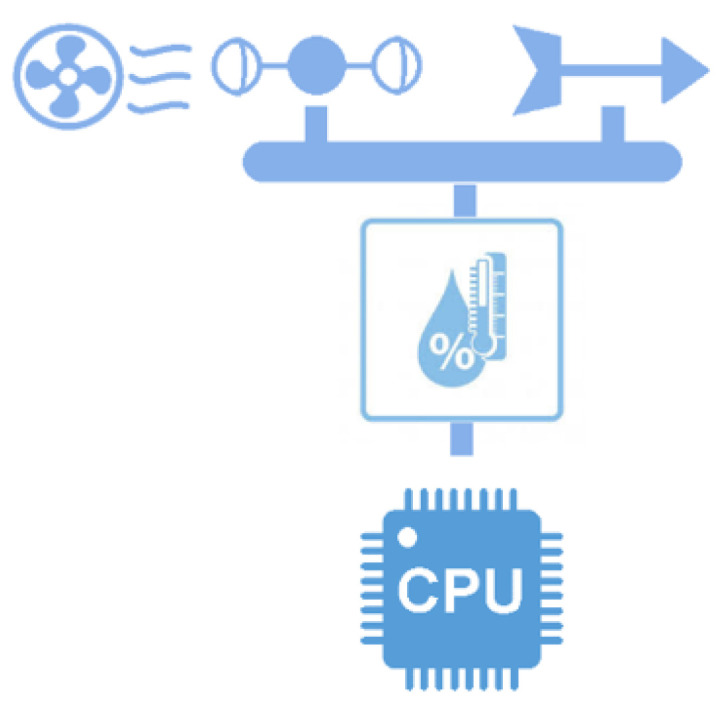
MEIoT weather station with exogenous disturbance input icon.

The MEIoT weather station comprises a microchip microcontroller containing a Control Processing Unit (CPU) to process the information. The microcontroller is connected to the sensors to receive the sensed data and a motor-based actuator that can be activated and controlled (speed and spin direction) through PWM. The microcontroller is also connected to the cloud to upload the sensed data via WiFi. Figure 6 depicts the icon used for MEIoT weather station, adding the exogenous disturbance input.

### 3.3. MEIoT Weather with Exogenous Disturbance Input Sensors and Actuator

Sensors are described in [17]. Each one of them was analyzed and selected considering their features and how suitable they are for the application and considering price and availability.

As it is mentioned in [17], the system requires three fundamental phases for its design and implementation process: cloud-based database design, communication between elements, and an open-source website platform.

The Relational Model developed for this system was modified to add the PWM duty cycle and the sampling time. The website platform can also consult the database.

**Figure 7:**
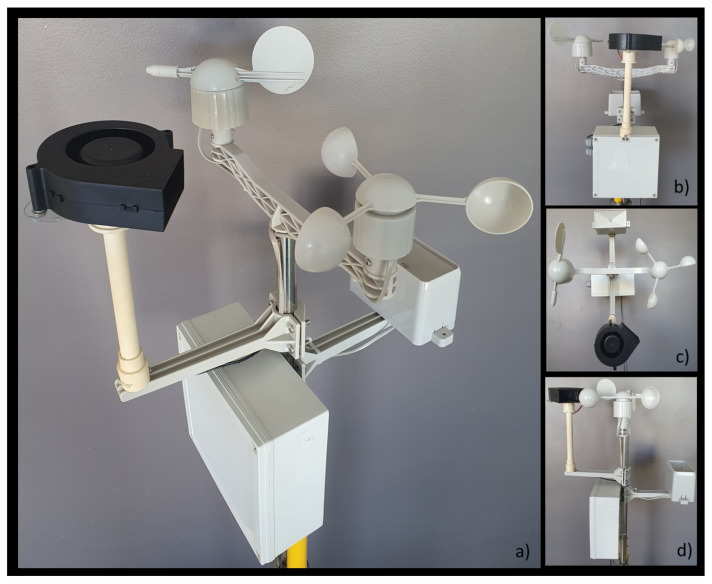
MEIoT weather station with exogenous disturbance input. (**a**) Isometric view, (**b**) front view, (**c**) top view and (**d**) right view.

We used an ESP32 microcontroller which had two xtensa 32-bits LX6 microprocessors in an Arduino based board; sensors are connected using the I2C communication bus, analog ports, and external interruptions. We used a dedicated PWM pin and two General Port Input-Output (GPIO) pins for direction and enable for driveing the exogenous disturbance. The embedded software was developed in C++ language, taking advantage of the dual-core. One core manages the sensors’ connections and measurements while it also establish the exogenous disturbance input. Once the sampling time was reached (10 s for this implementation), this core created a JSON document with all their information, and passed it to the second core. The second core managed the internet connection; it sent the JSON document to IBM Watson IoT through publishing it in a specific MQTT topic. This core also subscribed to a specific topic to retrieve commands in a JSON document format. The electronics were arranged in a small plastic perforated enclosure mounted on a tripod for practical usage (see Figure 7), and could be moved to a specific location for running tests in different environments.

Some different wind sources were analyzed for the exogenous disturbance input, a pedestal fan, a computer fan, Figure 4a, a mini desktop fan, Figure 4b, and an air blower, Figure 4c. One of the main features considered for selecting one was suitability, discarding the pedestal fan due to its dimensions, power consumption, a more complicated power stage, and higher price. The other three fans were very similar in their electrical characteristics, power stage, dimensions, and price.

A field test was carried out to select one from the 3 remaining fans. The selection criteria were that the fan must generate a wind stream strong enough to stimulate the anemometer and a speed range wide enough so that a 10% difference in the power output registered a notable difference in the sensor’s response. The air blower was the best option under the selection criteria.

**Figure 8:**
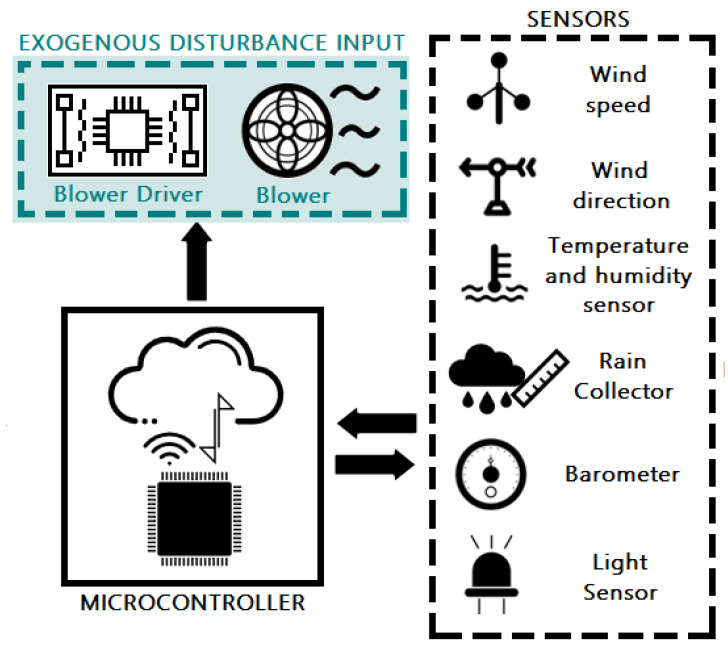
MEIoT weather station with exogenous disturbance input schematic.

A Radox 510-752 blower model was employed, Figure 4c. It had a 3.84 W max energy consumption at 12 V, with a maximum speed of 3000 Revolutions Per Minute (RPM). The microcontroller used an open loop control to drive a dedicated PWM from the microcontroller, Figure 8. Finally, an HW-95 board with an L298N dual H-Brigde was used as a power stage to drive the blower motor. This exogenous disturbance source can generate disturbances for both wind speed and wind direction sensors that can stimulate those registered inputs’ variability.

### 3.4. Graphic User Interface GUI-MEIoT 2.0

Several applications can be used for the visualization and manipulation of the data of an IoT application. For the online platform, in a preview work, we used Grafana Cloud [17]. However, we had to change to the Node-RED on IBM Cloud because Grafana only allowed us to receive data from devices, and it was not designed to send commands or information to devices. Node-RED is a programming tool for connecting devices, APIs, and online services, while Grafana is for online database queries and management. Node-RED is built on Node.js, so it has access to more than 225,000 modules in the Node’s package repository.

The navigation of the website is intuitive and easy for the user to use. The platform also allows setting the framing and the start and end time to show the later graphics. The user also can download the proportions from the website in a Comma-Separated Value (CSV) format.

With Node-RED, we can define different user roles to establish the resources each user can access. So far we have defined two roles Educator and User.

- Educator: Can visualize and interact with the dashboard and MEIoT weather station parameters, for example, measurement units, sampling time, delete database registers. This profile can not add, edit, or delete data sources.
- User: Can visualize and interact with the dashboard and data if they have access. They cannot delete database registers.

The sensed data are stored in a MySQL database. Node-RED also presents a set of tools for consultation that are useful for data visualization and analysis.

**Figure 9:**
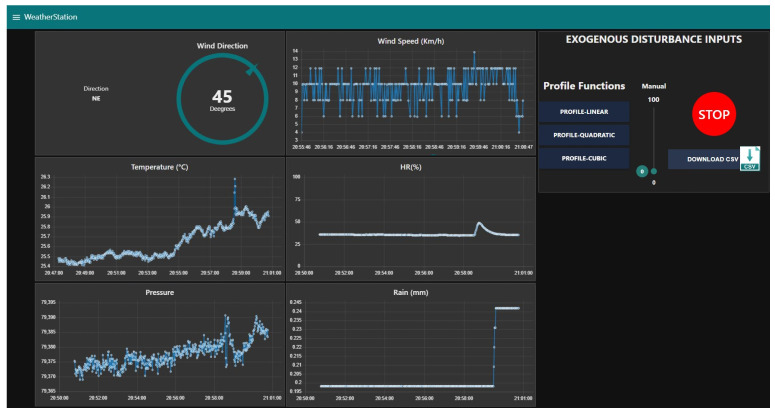
GUI-MEIoT 2.0 on Node-RED.

Figure 9 shows a dashboard screenshot from an 15 min experiment we made to test the sensors’ response. In the upper row, from left to right, we can see the wind direction and wind speed; in the middle row temperature and relative humidity; and the bottom row shows barometric pressure and pluvial precipitation. Finally the exogenous disturbance input control is shown in the screenshot’s upper right, where it can be controlled automatically via the profile functions or manually through the sliding bar. Light sensor measurement is not shown as it was not tested in our experiment.

## 4. Application to Education Engineering within the Educational Mechatronics Framework Using MEIoT Weather Station with Exogenous Disturbance Input

**Figure 10:**
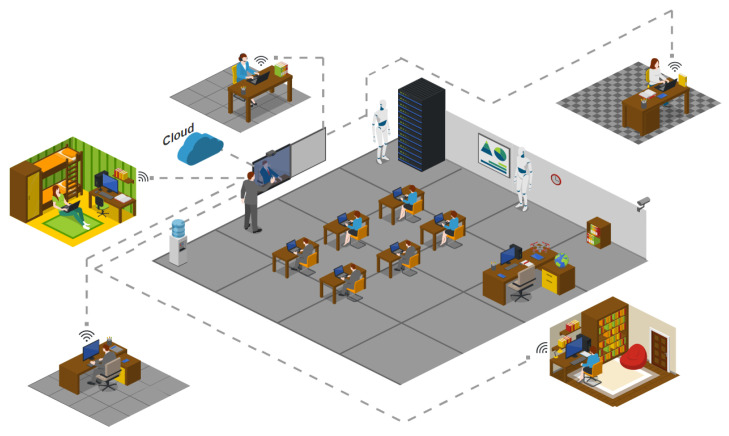
Hybrid Educational IoT Environment.

This work’s educational collaboration scenario involves educators and participants engaged in interactive and dynamic synchronous sessions simultaneously in person and online, the so-called hybrid learning (see Figure 10). The connection between various actors is managed and facilitated by the OBNiSE, from where issues such as access, data availability, access to the MEIoT station, data visualization, dashboard configuration, data modifications, among other things, can be made.

**Figure 11:**
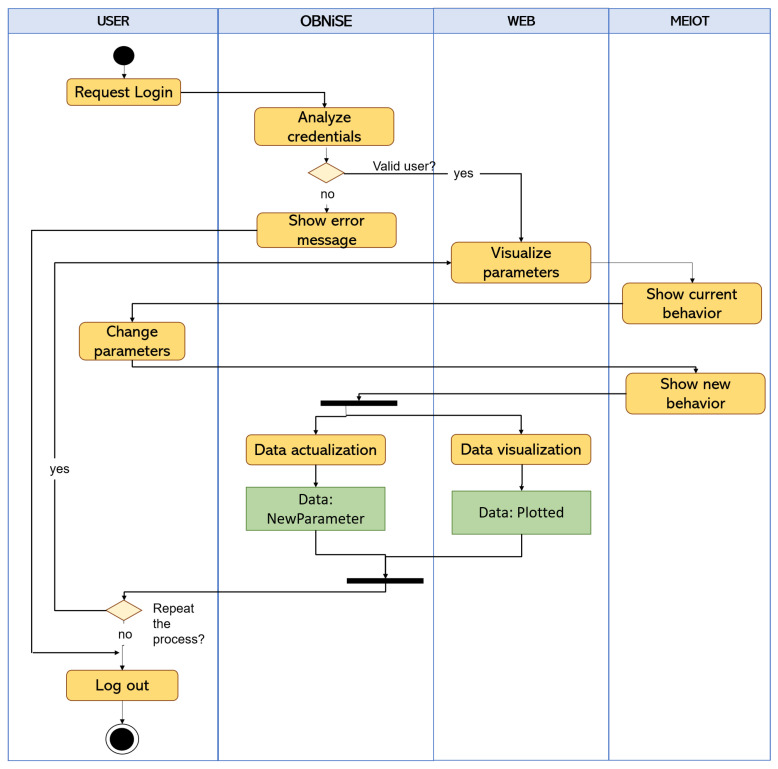
Activity diagram between participants, the web system, and the MEIoT weather station.

Each of the users (educator or participant) has an independent connection within the system that allows queries or modifications to the MEIoT weather station; the process that describes the connection and the visualization of the data is shown in Figure 11.

As it can be seen, a participant request access to the web through a link shared by the educator or using a username and password; once the credentials are validated, the Web system displays the current data of the MEIoT weather station. The participant can then observe the parameter or parameters to be modified; in this case it is only one parameter, the wind speed. The parameter is sent over the network to the server. Once the server responds to the request, it updates the sensor’s behavior and updates the data in the web system. The entire process of requesting and updating data is carried out in an estimated time of 5 min per participant request. The participant can modify the exogenous disturbance as many times as possible to observe the wind speed changes at the MEIoT station. The waiting time varies according to the number of users connected to the web. The server’s current operation is as follows, each time a request is received, it is queued and served in the order of arrival.

**Figure 12:**
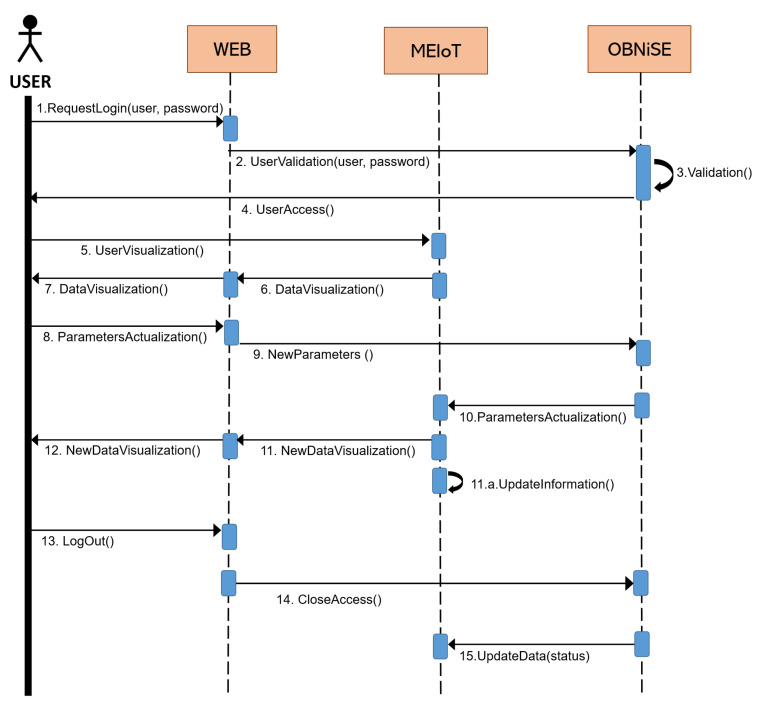
Sequential diagram connection between the user and the system.

The connection process between the users with the website and the weather station is described in Figure 12. The exchange of messages between the user’s device, the website, and the weather station allows the access and successful modification of the station parameters during the time the user is connected. The website allows students to visualize and understand the practical understanding, while the theoretical part is described in the next section. This model is adaptable to different scenarios and allows students to become familiar with technological platforms that enhance their knowledge with a practical part and a theoretical part that describes the applied knowledge. For this proposal, some knowledge, including Internet of Things, mechatronics, and algebra, to name a few, are considered.

The OBNiSE IoT architecture allows us to integrate and perform applications in several areas, such as Mobility, Health, Smart Cities, Technology, and Education. In particular, this proposal presents the application of engineering education that aims to develop skills and abilities required by the I4.0, and promote active learning using resources, existing academic spaces, practical activities, and mechatronic prototypes based on an innovative educational methodology: [30]. These courses represent an alternative to improve and reduce the gap between the current knowledge at schools and the new industry requirements. The instructional design based on the EMCF is presented below.

### Instructional Design

The instructional design is focused on the subject of least squares regression, and the linear, quadratic and cubic approximations are presented. The EMCF involves three perspective entities: statistics (process) + Internet of Things (application) + MEIoT weather station (artifact). The mechatronic concept is presented and defined in [31] as:

*“For a set of points $\left(x_{1},y_{1}\right)$,$\left(x_{1},y_{1}\right)$,...,$\left(x_{1},y_{1}\right)$, the least squares regression is given by*

*$f\left(x\right)=a_{0}+a_{1}x$ (linear approximation)*

*$f\left(x\right)=a_{0}+a_{1}x+a_{2}x^{2}$ (quadratic approximation)*

*$f\left(x\right)=a_{0}+a_{1}x+a_{2}x^{2}+a_{3}x^{3}$ (cubic approximation)*

*that minimizes the sum of the squared error*

${\left[y_{1}-f\left(x_{1}\right)\right]}^{2}+{\left[y_{2}-f\left(x_{2}\right)\right]}^{2}+\ldots +{\left[y_{n}-f\left(x_{n}\right)\right]}^{2}$.”

The pedagogical activities for the three levels with the selected perspective, which are concrete learning level, graphic learning level, and abstract learning level, are defined as follows. First, the educator turns on the MEIoT weather station with exogenous disturbance input to go through the session, and all the participants log in to the web system.

**Figure 13:**
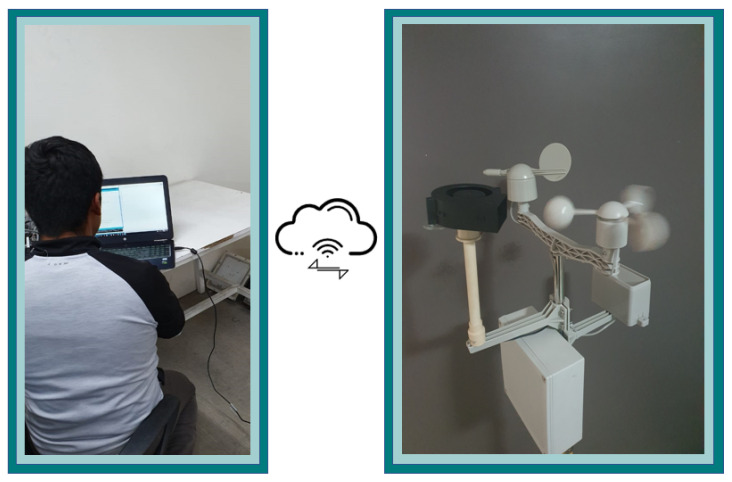
Concrete level: MEIoT weather station.

**Figure 14:**
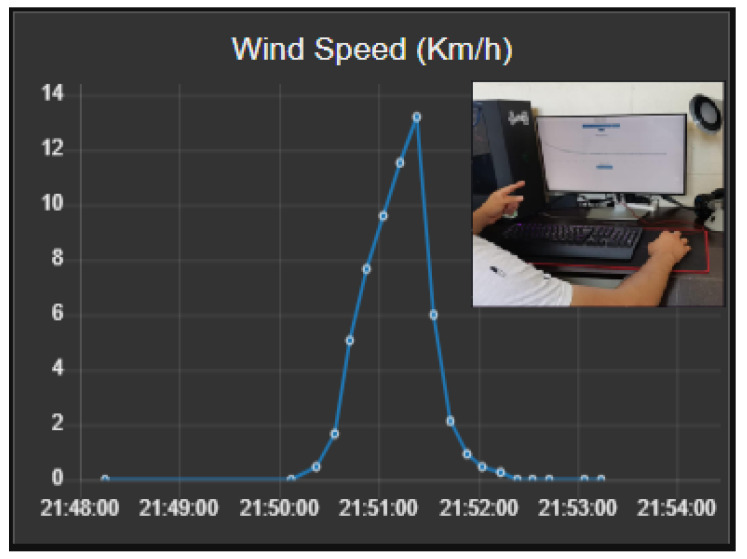
Instructional design: graphic level showing the weather station’s data in real-time with the open-source platform.

**Figure 15:**
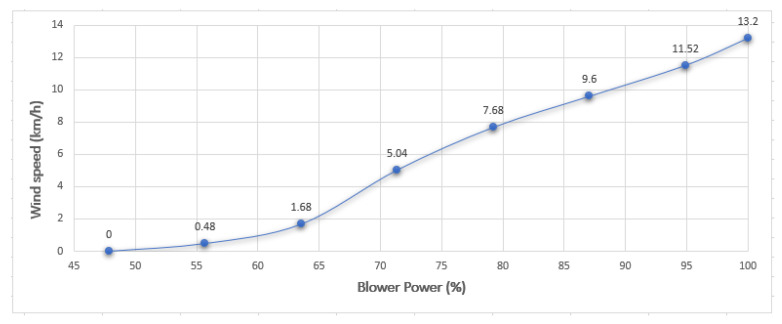
Instructional design: graphic level showing the blower power vs measured wind speed plot.

**Figure 16:**
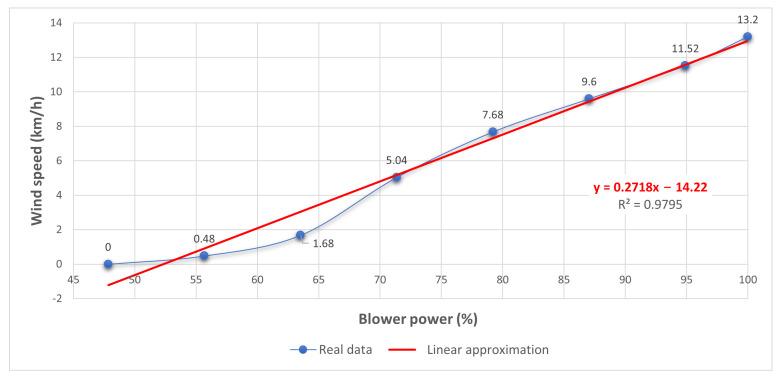
Instructional design: abstract level.

**Figure 17:**
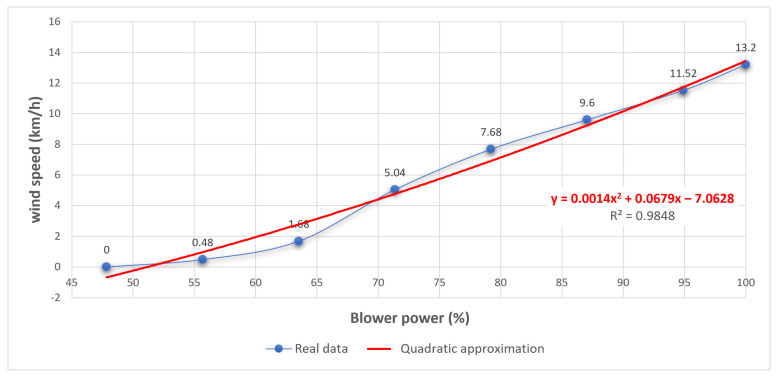
Quadratic regression vs. real data.

**Figure 18:**
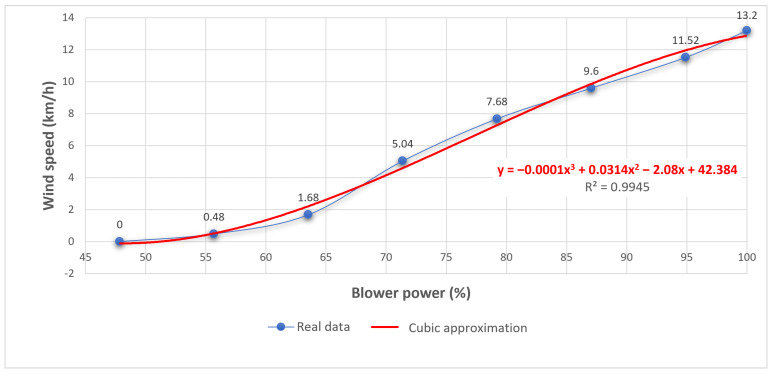
Cubic regression vs real data.

- Concrete Learning Level (CLL). At this level, activities aimed at perceptual-motor characteristics should be designed using the MEIoT Weather Station with exogenous disturbance input (See Figure 13). The activities to perform at this level, considering only the wind speed variables at 10 s of sampling time, are described as follows.
  1. The participant moves the slider “Manual” control from 0% to 100% to manually change the exogenous disturbance input of the MEIoT weather station and observe what is happening with the wind speed chart. Finally, click on the “STOP” button.
  2. The participant send a linear profile to the MEIoT weather station by clicking the “Profile-linear” button.
- Graphic Learning Level (GLL). At this level, activities aimed at the graphic (symbolic) representation of mechatronic concepts should be designed, taking reference to the concepts learned previously at the concrete learning level. The learning will gradually make the transition from the concrete to the abstract level. For the graphical level to be more significant, the online open-source platform Node-RED is used to display the wind speed collected data. The participant can visualize the sensor’s wind speed dynamics and how wind speed value is increasing as time passes until reaching the final point of the introduced profile (see Figure 14). Tasks related to this level are described below.
  1. The participant sends a linear profile to the MEIoT weather station by clicking in the “Profile-linear” button.
  2. The participant visualizes the GUI-MEIoT to observe the past values and current value of the wind speed variable.
  3. In a white paper sheet, the participant plots each wind speed value with points.
  4. Starting from the first point and linking all points using smooth lines.
  5. Download the .CSV file by clicking in the download icon. *Remark:* Here the participant moves the pencil to link all the points. Figure 15 depicts the resulting plot.
  The data collection registered by the participant is shown in (Table 1).
- Abstract Learning Level (ALL). At this level, activities should be designed to gradually transition from symbolic concepts to abstract representation that includes mathematical equations. In many problems in the biological, physical, social sciences, and engineering, it is useful to describe the relationship between the same variables through a mathematical expression. A common way to do this is to adjust a curve between the various data points. This curve can be linear or quadratic or cubic, and so on. The goal is to find the curve of the specific type that fits “best” to the given n−data points comprising in the introduced linear profile (see Table 1), where $x_{i}=$ blower power, $y_{i}=$ wind speed (km/h) and $f\left(x_{i}\right)=a_{0}+a_{1}x_{i}=$ value of the approximation in $x_{i}$.
  To find the least-squares regression line for a set of points, begin by forming the system of linear equations
  $$\begin{array}{c} y_{1}=f\left(x_{1}\right)+\left[y_{1}-f\left(x_{1}\right)\right]\\ y_{2}=f\left(x_{2}\right)+\left[y_{2}-f\left(x_{2}\right)\right]\\ \vdots \\ y_{n}=f\left(x_{n}\right)+\left[y_{n}-f\left(x_{n}\right)\right] \end{array}$$
  where the right-hand term, $\left[y_{i}-f\left(x_{i}\right)\right]$, of each equation is thought of as the error in the approximation of $y_{i}$ by $f(x_{i})$. Then writing this error as $e_{i}=y_{i}-f\left(x_{i}\right)$, we yield
  (1) $$\begin{array}{c} y_{1}=\left(a_{0}+a_{1}x_{1}\right)+e_{1}\\ y_{2}=\left(a_{0}+a_{1}x_{2}\right)+e_{2}\\ \vdots \\ y_{n}=\left(a_{0}+a_{1}x_{n}\right)+e_{n} \end{array}$$
  Now, we define
  (2) $$y=\left(\begin{array}{c} y_{1}\\ y_{2}\\ \vdots \\ y_{n} \end{array}\right),\;A=\left(\begin{array}{cc} 1 & x_{1}\\ 1 & x_{1}\\ \vdots & \vdots \\ 1 & x_{n} \end{array}\right),\;u=\left(\begin{array}{c} a_{0}\\ a_{1} \end{array}\right),\;e=\left(\begin{array}{c} e_{1}\\ e_{2}\\ \vdots \\ e_{n} \end{array}\right)$$
  Then, the matrix form for linear approximation is given by Equation (3)
  (3) y=Au+e
  solving the variable *u* from the equation we obtain the values of the coefficients $a_{0}$ and $a_{1}$ of the least squares approximation line as
  (4) $$u={\left(A^{T}A\right)}^{-1}A^{T}y$$
  To find the line to best fits the points, first the mathematical objects has to be formed as it is presented in Equation (3).
  $$y=\left[\begin{array}{c} 0\\ 0.48\\ 1.68\\ 5.04\\ 7.68\\ 9.6\\ 11.52\\ 13.2 \end{array}\right],A=\left[\begin{array}{cc} 1 & 47.84313725\\ 1 & 55.68627451\\ 1 & 63.52941176\\ 1 & 71.37254902\\ 1 & 79.21568627\\ 1 & 87.05882353\\ 1 & 94.90196078\\ 1 & 100 \end{array}\right]$$
  $$\begin{array}{c} u={\left(A^{T}A\right)}^{-1}A^{T}y \end{array}$$
  $$u={\left({\left[\begin{array}{cc} 1 & 47.84313725\\ 1 & 55.68627451\\ 1 & 63.52941176\\ 1 & 71.37254902\\ 1 & 79.21568627\\ 1 & 87.05882353\\ 1 & 94.90196078\\ 1 & 100 \end{array}\right]}^{T}\left[\begin{array}{cc} 1 & 47.84313725\\ 1 & 55.68627451\\ 1 & 63.52941176\\ 1 & 71.37254902\\ 1 & 79.21568627\\ 1 & 87.05882353\\ 1 & 94.90196078\\ 1 & 100 \end{array}\right]\right)}^{-1}{\left[\begin{array}{cc} 1 & 47.84313725\\ 1 & 55.68627451\\ 1 & 63.52941176\\ 1 & 71.37254902\\ 1 & 79.21568627\\ 1 & 87.05882353\\ 1 & 94.90196078\\ 1 & 100 \end{array}\right]}^{T}\left[\begin{array}{c} 0\\ 0.48\\ 1.68\\ 5.04\\ 7.68\\ 9.6\\ 11.52\\ 13.2 \end{array}\right]$$
  $$\begin{array}{c} u=\left[\begin{array}{c} 14.220\\ 0.27178 \end{array}\right] \end{array}$$
  The linear approximation that best fits the points is y=0.27178x−14.220.
  Now, we can implement this in Excel for both the real wind speed data and the obtained linear approximation (see Figure 16). Moreover, Table 2 shows the sum of the squared errors made in the results of every single data and also the total sum of 3.7793.
  Now, we have to find the best quadratic fit for the points. In order to do so, we have to define the following equation as in Equation (1).
  (5) $$\begin{array}{c} y_{1}=\left(a_{0}+a_{1}x_{1}+a_{2}x_{1}^{2}\right)+e_{1}\\ y_{2}=\left(a_{0}+a_{1}x_{1}+a_{2}x_{1}^{2}\right)+e_{2}\\ \vdots \\ y_{n}=\left(a_{0}+a_{1}x_{n}+a_{2}x_{n}^{2}\right)+e_{n} \end{array}$$
  Then, we formed the vectors as the ones presented in Equation (2) and solving for *u* we yield to.
  (6) $$\begin{array}{c} y=\left[\begin{array}{c} y_{1}\\ y_{2}\\ y_{3}\\ y_{4}\\ y_{5}\\ y_{6}\\ y_{7}\\ y_{8} \end{array}\right]=\left[\begin{array}{c} 0\\ 0.48\\ 1.68\\ 5.04\\ 7.68\\ 9.6\\ 11.52\\ 13.2 \end{array}\right],A=\left[\begin{array}{ccc} 1 & x_{1} & x_{1}^{2}\\ 1 & x_{2} & x_{2}^{2}\\ 1 & x_{3} & x_{3}^{2}\\ 1 & x_{4} & x_{4}^{2}\\ 1 & x_{5} & x_{5}^{2}\\ 1 & x_{6} & x_{6}^{2}\\ 1 & x_{7} & x_{7}^{2}\\ 1 & x_{8} & x_{8}^{2} \end{array}\right]=\left[\begin{array}{ccc} 1 & 47.84313725 & 47.843137252^{2}\\ 1 & 55.68627451 & 55.68627451^{2}\\ 1 & 63.52941176 & 63.52941176^{2}\\ 1 & 71.37254902 & 71.37254902^{2}\\ 1 & 79.21568627 & 79.21568627^{2}\\ 1 & 87.05882353 & 87.05882353^{2}\\ 1 & 94.90196078 & 94.90196078^{2}\\ 1 & 100 & 100^{2} \end{array}\right]\\ \;\;u={\left(A^{T}A\right)}^{-1}A^{T}y=\left[\begin{array}{c} -7.0628\\ 6.7895x10^{-2}\\ 1.3717x10^{-3} \end{array}\right] \end{array}$$
  The quadratic approximation function that best fits the points is $y=0.0013717x^{2}+0.067895x-7.0628$ (see Figure 17). This approximation leads to a total sum of squared errors of 2.7989.
  Follow the same procedure for finding the best cubic fit for the points.
  (7) $$\begin{array}{c} y_{1}=\left(a_{0}+a_{1}x_{1}+a_{2}x_{1}^{2}+a_{3}x_{1}^{3}\right)+e_{1}\\ y_{2}=\left(a_{0}+a_{1}x_{1}+a_{2}x_{1}^{2}+a_{3}x_{2}^{3}\right)+e_{2}\\ \vdots \\ y_{n}=\left(a_{0}+a_{1}x_{n}+a_{2}x_{n}^{2}+a_{3}x_{n}^{3}\right)+e_{n} \end{array}$$
  Then, we formed the vectors as the ones presented in Equation (2) and solving for *u* we yield to
  (8) $$y=\left[\begin{array}{c} y_{1}\\ y_{2}\\ y_{3}\\ y_{4}\\ y_{5}\\ y_{6}\\ y_{7}\\ y_{8} \end{array}\right],A=\left[\begin{array}{cccc} 1 & x_{1} & x_{1}^{2} & x_{1}^{3}\\ 1 & x_{2} & x_{2}^{2} & x_{2}^{3}\\ 1 & x_{3} & x_{3}^{2} & x_{3}^{3}\\ 1 & x_{4} & x_{4}^{2} & x_{4}^{3}\\ 1 & x_{5} & x_{5}^{2} & x_{5}^{3}\\ 1 & x_{6} & x_{6}^{2} & x_{6}^{3}\\ 1 & x_{7} & x_{7}^{2} & x_{7}^{3}\\ 1 & x_{8} & x_{8}^{2} & x_{8}^{3} \end{array}\right]$$
  (9) $$u={\left(A^{T}A\right)}^{-1}A^{T}y=\left[\begin{array}{c} 42.384\\ -2.08\\ 0.03139\\ -1.3541x10^{-4} \end{array}\right]$$
  The cubic approximation function that best fits the points is $y=-0.00013541x^{3}+0.3139x^{2}-2.08x+42.384$ (see Figure 18). This approximation leads to a total sum of squared errors of 1.0203.
  Finally, the obtained total sums of squared errors for the linear, quadratic and cubic approximations has to be compared. It can be noted that the cubic approximation is the best fit for the real data.

**Figure 19:**
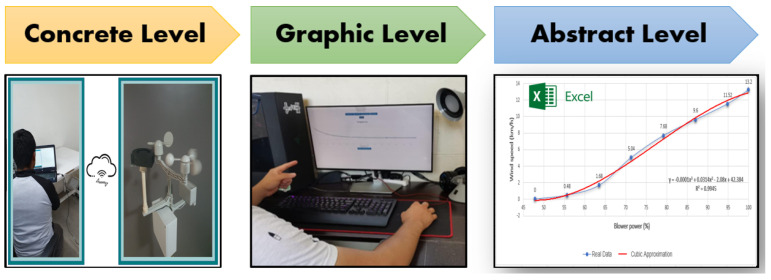
Complete instructional design.

As a summary, Figure 19 shows the complete instructional design with the three main levels applying the EMCF.

It is worth mentioning that the concrete level presenting the process to build the MEIoT weather station could be followed by any person with an engineering background and understand the required hardware. The open-source software is easy to use and understand for users; only simple configurations to the model are required. Finally, the proposal describes the least-squares regression procedure at the abstract level that can be easily extended for more polynomial functions and applications.

## 5. Discussion

This novel MEIoT weather station with exogenous disturbance input has been designed and developed as a compact device that allows monitoring weather variables and generates an input perturbance every 10 s in a defined period. The integration of an actuator to disturb a sensor input allows the interaction demanded by hands-on learning. Moreover, this weather station can be easily adapted to any environment without expensive infrastructure. Its conformance to the OBNiSE IoT architecture allows it to be securely accessed from any connected part of the world.

While the proposed configuration does not yield a linear response to the generated exogenous disturbance input, this can be solved with the controller configuration. Additionally, only one exogenous disturbance was generated for this work, but it was enough to prove that an exogenous disturbance input can help manipulate the monitored system state. The exogenous disturbance input can be varied for the wind speed sensor. However, its construction limits it for a fixed disturbance in the wind direction sensor, so the wind direction sensor response was not considered in this work.

Compared with other works, such as [12] where the data is stored locally. Alternatively, in [13,15], the goal was only measured data availability. In [16] uses ZigBee to transmit the station data wirelessly to a local display. Our proposal adapts well to engineering education by adding an interaction layer that is not found in other works.

The Educational Mechatronics Framework guided us to apply the three learning construction levels, concrete, graphic, and abstract, and cover them with an application to education engineering that uses the MEIoT weather station with exogenous disturbance input as an artifact. In this sample application, the students can infer how to fit any polynomial curve to a data set using least square regression and analyze the better fit. We expect students to achieve or even overachieve their grades by applying this online experimentation tool, as [3] reported with their online education scheme.

This novel MEIoT weather station with exogenous disturbance input within the OBNiSE IoT architecture has been tested with a pilot test. It is poised to be deployed with engineering students for a HOL approach in a remote access laboratory or hybrid education environment. The change from a database visualizer like Grafana [13,17] to a programming environment like Node-RED gave us more control for running experiments remotely, which several weather stations can not do.

Our approach also represents a very low-cost investment for remote access laboratory equipment. While local sensor data capture and transmission is done in an MCU, the cloud processing is done with IBM cloud services; depending on the demand, a free lite service may be enough, and the GUI is developed in an open-source platform. Other approaches like [10], where the system is based on PLC and SCADA, or [11], where their user interface is developed in LabVIEW, involves high licenses cost or significant infrastructure investment. In [9], where a PC does the control interface with a LabVIEW program and user interface is running in an Apache web server, can also mean a high energy cost to have complete availability.

The application of this MEIoT weather station with exogenous disturbance input in the educational environment and this pandemic situation due to SARS-CoV-2 will allow students to attend laboratory classes while respecting the isolation measures, as suggested by [1]. Once the pandemic passes, this development will remain a useful tool by enabling hybrid education by allowing students in remote locations to conduct guided laboratory practices in an environment that is not just a computer simulation. It also allows the student’s independent work with laboratory equipment with full availability to further complement their education. Finally, this type of development can significantly appeal to educational institutions with several campuses by allowing a piece of laboratory equipment that can be remotely shared throughout the institution without moving the equipment or the students and advisors.

Future work will involve integrating more exogenous disturbance inputs besides the one showcased here, such as heat, light, humidity, and sources. Additionally, to implement a moving base for the air blower to control the wind direction sensor’s disturbance. Furthermore, tune the open-loop controller to achieve a linear response from the sensor-actuator interaction. Integrate video streaming to see in real-time the effects of the exogenous disturbance inputs in the sensors. Finally, create more education engineering applications red based on the MEIoT weather station with exogenous disturbance inputs.

## Figures and Tables

**Table 1:** Data collection of the wind speed measured by the participant.

Blower Power (%)	Wind Speed (km/h)
47.84313725	0
55.68627451	0.48
63.52941176	1.68
71.37254902	5.04
79.21568627	7.68
87.05882353	9.60
94.90196078	11.52
100.00000000	13.20

**Table 2:** Sum of squared errors.

$x_{i}$ = Blower Power (%)	$y_{i}$ = Wind Speed (km/h)	$f(x_{i})$	${\left[y_{i}-f\left(x_{i}\right)\right]}^{2}$
47.84313725	0	−1.2172	${\left(1.2172\right)}^{2}$
55.68627451	0.48	0.91442	${\left(-0.43442\right)}^{2}$
63.52941176	1.68	3.0460	${\left(-1.366\right)}^{2}$
71.37254902	5.04	5.1776	${\left(-0.1376\right)}^{2}$
79.21568627	7.68	7.3092	${\left(0.3708\right)}^{2}$
87.05882353	9.60	9.4408	${\left(0.1592\right)}^{2}$
94.90196078	11.52	11.572	${\left(-0.052\right)}^{2}$
100.00000000	13.20	12.958	${\left(0.242\right)}^{2}$
Total			3.7793

## Data Availability

The data presented in this study are available in Table 2.

## Abbreviations

The following abbreviations are used in this manuscript:

ADC	Analog-to-digital Converter
ALL	Abstract Learning Level
API	Application Program Interface
CIIDETEC-UVM	Centro de Investigación, Innovación y Desarrollo Tecnológico, UVM
CMOS	Complementary Metal Oxide Semiconductor
CLL	Concrete Learning Level
COVID 19	Coronavirus Disease 2019
CPU	Central Processing Unit
CSV	Comma-Separated Values
EMCF	Educational Mechatronics Conceptual Framework
GLL	Graphic Learning Level
GPIO	General Port Input-Output
GUI	Graphic User Interface
HOL	Hands-on learning
IC	Integrated Circuit
ICT	Information and communication Technology
I4.0	Industry 4.0
IoT	Internet of Things
I2C	Inter-Integrated Circuit
KPI	Key Performance Indicators
MEIoT	Mechatronic Educational Internet of Things
MEMS	Microelectromechanical Systems
MCU	Microcontroller Unit
MQTT	Message Queuing Telemetry Transport
OBNiSE	Observatorio Nacional Digital de Smart Environments
P2P	Peer-to-peer
PWM	Pulse Width Modulator
RPM	Revolutions Per Minute
RoHS	Restriction of Hazardous Substances
SCADA	Supervisory Control And Data Acquisition
SMD	Surface Mount Device
TLS	Transport Layer Security
UniMAP	University of Malaysia Perlis
UVM	Universidad del Valle de México
VNC	Virtual Network Computing
